# Monocusp patch from bovine jugular vein valved graft (Contegra®) for right ventricular outflow recostruction in tetralogy of fallot repair

**DOI:** 10.1186/1749-8090-8-S1-O311

**Published:** 2013-09-11

**Authors:** Fotios Mitropoulos, Andrew C Chatzis, Meletios A Kanakis, Constantinos Contrafouris, Michael Milonakis, Nicholaos Giannopoulos, Alexander Tsoutsinos, Prodromos Azariadis

**Affiliations:** 1 Department of Paediatric and Congenital Cardiac Surgery, Onassis Cardiac Surgery Centre, Kallithea 17674, Athens, Greece; 2 Department of Paediatric Cardiology, Onassis Cardiac Surgery Centre, Kallithea 17674, Athens, Greece

## Objective

Absence of a competent pulmonary valve (PV) after tetralogy of Fallot repair bears severe impact on right ventricular (RV) function postoperatively and the construction of a monocusp valve substitute has been recommended. We have used a Contegra® graft for this purpose and present herein the results of its use.

## Methods

From July 2007 to September 2012, 24 patients, 14 males and 10 females age 5 months to 5.5 years (median 13 months) and median BSA 0.423m2 underwent tetralogy of Fallot repair requiring extended transannular incision to relieve right ventricular outflow tract (RVOT) obstruction. Reconstruction was achieved utilising part of a Contegra® graft containing one of the cusps (monocusp).

## Results

There were no deaths. Median ICU and hospital stay were 5 and 11 days respectively. Median postoperative (discharge) PV peak gradient (PG) was 23mmHg, median pulmonary regurgitation (PR) 2+/4+ and median tricuspid regurgitation (TR) 1+/4+. Ten patients were followed up for a median of 13 months. For this particular group of patients postoperative vs. follow-up measurements (median values): PV-PG; 20 mmHg vs. 21mmHg, PR; 2+/4+ vs. 2+/4+, TR; 1+/4+ vs. 1+/4+ respectively.

## Conclusion

The use of a monocusp valve derived from a Contegra® graft achieves acceptable PR, preserving thus RV function with good overall immediate postoperative and short term results.

